# Retraction Note: Facial expressions can detect Parkinson’s disease: preliminary evidence from videos collected online

**DOI:** 10.1038/s41746-023-00761-7

**Published:** 2023-02-03

**Authors:** Mohammad Rafayet Ali, Taylor Myers, Ellen Wagner, Harshil Ratnu, E. Ray Dorsey, Ehsan Hoque

**Affiliations:** 1 Computer Science, University of Rochester, Rochester, NY USA; 2 Center for Health+Technology, University of Rochester Medical Center, Rochester, NY USA

**Keywords:** Parkinson's disease, Computer science

Retraction to: *npj Digital Medicine* 10.1038/s41746-021-00502-8, published online 03 September 2021

The authors have retracted this article because the classification described in the paper was performed on an inaccurate use of a data pre-processing tool, which resulted in an information leak from train to test samples. This has resulted in incorrect classification metrics. However, the statistical differences between features of PD and non-PD populations indicate that this is still an area worthy of further investigation. All authors agree with this retraction.

